# Medullary Carcinoma of the Colon: A Case Report

**DOI:** 10.7759/cureus.105818

**Published:** 2026-03-25

**Authors:** Marco Fabricio Bombón, Diego A Morales-Cisneros, Maria Eduarda Ubilla, Guido Panchana Coello, Lissette Estefanía García

**Affiliations:** 1 Surgical Oncology, Sociedad de Lucha Contra el Cáncer (SOLCA) Matriz, Instituto Oncológico Nacional “Dr. Juan Tanca Marengo”, Guayaquil, ECU; 2 General Surgery, Universidad de Especialidades Espíritu Santo (UEES), Guayaquil, ECU; 3 Surgery, Hospital General Guasmo Sur, Guayaquil, ECU

**Keywords:** colon neoplasm, digestive surgery, medullary colon cancer, right hemicolectomy, surgery

## Abstract

Medullary colon carcinoma (MCC) is a rare neoplasm that differs from conventional adenocarcinoma in clinical, histological, and molecular aspects. We present the case of a 68-year-old man with no significant medical history. Physical examination revealed a distended abdomen, painful on superficial and deep palpation. A pelvic and abdominal computed axial tomography (CT) scan with S/C IV contrast reported a solid tumor lesion with lobulated contours, located intraluminally within the cecum and part of the ascending colon, measuring 80 mm. The patient underwent exploratory laparotomy and right hemicolectomy, revealing a tumor of the ascending colon approximately 8 cm in diameter, involving the cecum and proximal region of the ascending colon. The histopathological and immunophenotypic report revealed a medullary carcinoma of the colon. Postoperative recovery was uneventful, and the patient remains asymptomatic during follow-up.

Given the low frequency of this neoplasm, this case highlights an appropriate approach and management based on scientific evidence, taking into account that timely and definitive surgical treatment allows tumor excision with adequate oncological margins, which can be complemented with adjuvant chemotherapy in advanced stages.

## Introduction

Medullary colon carcinoma (MCC) is a rare histological variant of colorectal adenocarcinoma, recognized by the World Health Organization (WHO) as a distinct entity due to its particular morphological and immunophenotypic characteristics. It accounts for less than 0.1% of colorectal cancers, predominantly affecting elderly women and occurring more frequently in the right colon, although it can occur in any segment of the colon [1,2]. Histologically, it is characterized by solid sheets of large tumor cells, with abundant eosinophilic cytoplasm, vesicular nuclei, and prominent nucleoli, accompanied by a prominent inflammatory infiltrate in the stroma [3].

Unlike other poorly differentiated adenocarcinomas, MCC is often associated with microsatellite instability (MSI-H) and mismatch repair deficiency (dMMR), which gives it a peculiar biological behavior and, in some cases, a better prognosis, despite its high histological grade [4,5]. The clinical presentation is nonspecific and may include common symptoms, such as abdominal pain, changes in bowel habits, gastrointestinal bleeding, or anemia, making early diagnosis difficult [6].

The definitive diagnosis requires a comprehensive approach, combining clinical findings, endoscopic and histopathological studies, and immunohistochemistry [7]. Typical immunohistochemical markers include positivity for cytokeratin 20 (CK20) and caudal-type homeobox 2 (CDX2) in a subgroup of cases, as well as frequent negativity for cytokeratin 7 (CK7); the expression profile may vary and should be interpreted in conjunction with morphology [8].

The treatment of choice is surgery, supplemented with adjuvant chemotherapy in advanced stages or with lymph node involvement [9]. Given the low frequency of this neoplasm, evidence on its management comes mainly from case reports and small series, which limits the existence of specific guidelines for its management [10].

This case report describes the clinical presentation, diagnosis, and treatment administered following histopathological confirmation of medullary carcinoma of the colon, taking into account the latest literature to guide diagnosis and appropriate, specific therapeutic management.

## Case presentation

A 68-year-old male patient, with a diagnosis of hypertension under treatment and a history of poorly differentiated colon adenocarcinoma confirmed in the pathology report following a previous colonoscopy (without specific cancer treatment), came to the Emergency department of this health center with severe abdominal pain, abdominal distension, loss of appetite, and nausea leading to vomiting on five occasions during the previous 12 hours. Physical examination revealed a distended abdomen, painful on both superficial and deep palpation. Additional tests were requested, including laboratory tests and a pelvic and abdominal computed axial tomography (CT) scan with S/C IV contrast.

Previous colonoscopy revealed evidence in the ascending colon of a vegetative, friable lesion of hard consistency, with the pathology report indicating poorly differentiated infiltrating adenocarcinoma. Paraclinical studies showed: GB: 4.28 × 10³/µL; HB: 8.6 g/dL; HCT: 30%; NEU: 75%; PLQ: 217 × 10³/µL, with other laboratory values within acceptable parameters. Tumor markers were: cancer antigen 19-9 (CA 19-9): 16.22; cancer antigen 72-4 (CA 72-4): 2.55; carcinoembryonic antigen: 3.16 (Table 1).

**Table 1:** Clinical laboratory tests on admission

Parameter	Result	Reference range
White blood cell count (WBC)	4.28 × 10³/µL	4,000-11,000/µL
Neutrophils	75%	40-75%
Hemoglobin	8.6 g/dL	13.5-17.5 g/dL
Hematocrit	30%	41-50%
Platelet count	4.28 × 10³/µL	150,000-450,000/µL
Serum sodium	135 mmol/L	135-145 mmol/L
Serum potassium	3.57 mmol/L	3.5-5.1 mmol/L
Serum chloride	100 mmol/L	98-106 mmol/L
Carcinoembryonic antigen	3.16 ng/mL	-
Cancer antigen 19-9 (CA 19-9)	16.22 U/mL	0-37 U/mL
Cancer antigen 72-4 (CA 72-4)	2.55 U/mL	<6.0 U/mL

**Figure 1:**
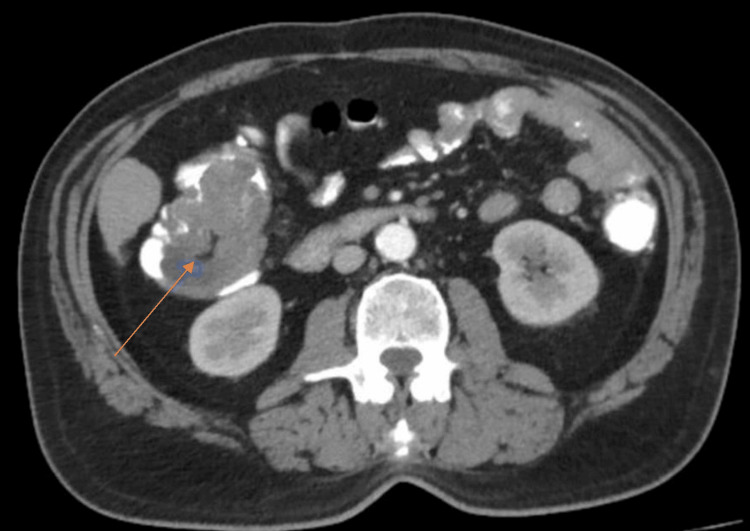
Axial computed tomography scan showing a solid tumor lesion with lobulated contours, located intraluminally within the cecum and part of the ascending colon (arrow)

CT with IV contrast reported a solid tumor lesion with lobulated contours, located intraluminally within the cecum and part of the ascending colon, measuring approximately 80 mm in its longitudinal axis, and associated with invagination of the distal ileum, cecal appendix, and adjacent mesenteric lymph nodes with morphological alteration (Figure 1).

The patient underwent surgery under general anesthesia: exploratory laparotomy, right hemicolectomy, D2 lymphadenectomy, and appendectomy, with the following findings: a tumor of the ascending colon, approximately 8 cm in diameter, involving the cecum (ileocecal valve); lymphadenopathy in the mesocolon, the largest measuring 1 cm, at the level of the right branch of the middle colic artery.

The final diagnosis was a poorly differentiated malignant neoplasm of epithelial appearance, ulcerated and exophytic, located in the ascending colon and involving the cecum, measuring 7.7 × 6 cm, with surgical margins free of malignancy. Two lymph nodes were positive for malignancy (2/40), while the cecal appendix showed no evidence of neoplasia or malignancy.

Immunohistochemical tests were also performed, with the following results: CK7, positive in neoplastic cells; CK20, negative in neoplastic cells; CDX2, heterogeneously positive in neoplastic cells; calretinin, positive in neoplastic cells; MutS homolog 6 (MSH6), intact in neoplastic cells; MutS homolog 2 (MSH2), intact in neoplastic cells; and MutL homolog 1 (MLH1), showing loss of expression in neoplastic cells.

**Figure 2:**
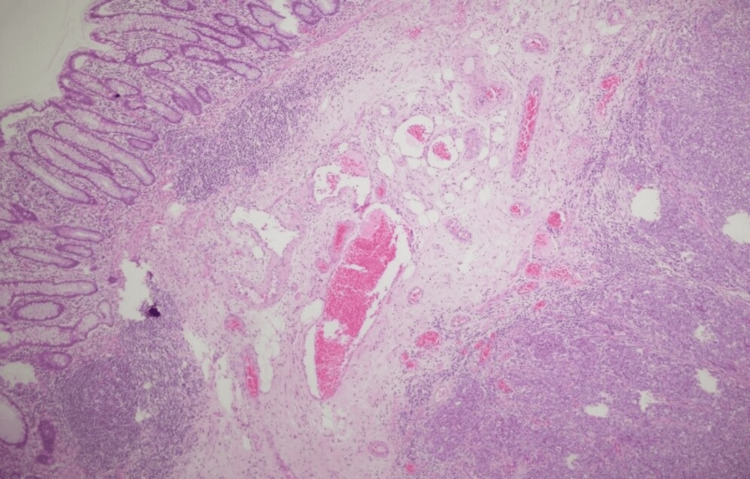
Microscopy: solid sheets of large, poorly differentiated tumor cells, with prominent nucleoli, are evident

**Figure 3:**
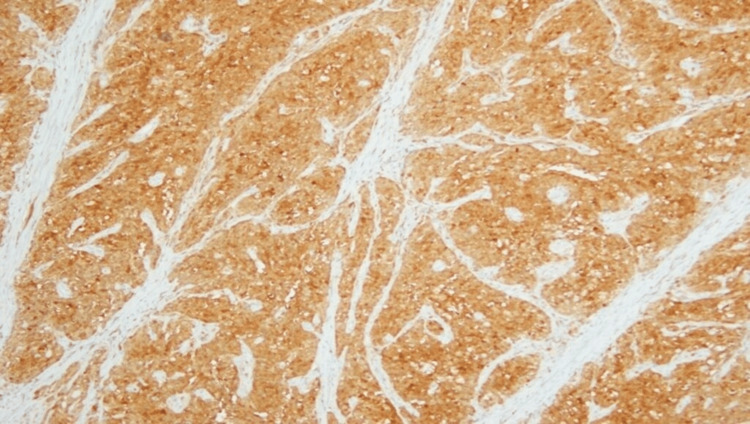
Microscopy: positive staining for calretinin is observed

The histopathological and immunophenotypic picture supports a diagnosis of medullary carcinoma of the colon, pT3N1b (Figures 2-3).

The procedure was completed without complications, with minimal bleeding. The patient was discharged six days after surgery, tolerating food, passing stool, and with an abdominal drain showing decreasing serosanguineous discharge, which was removed during an outpatient visit.

**Figure 4:**
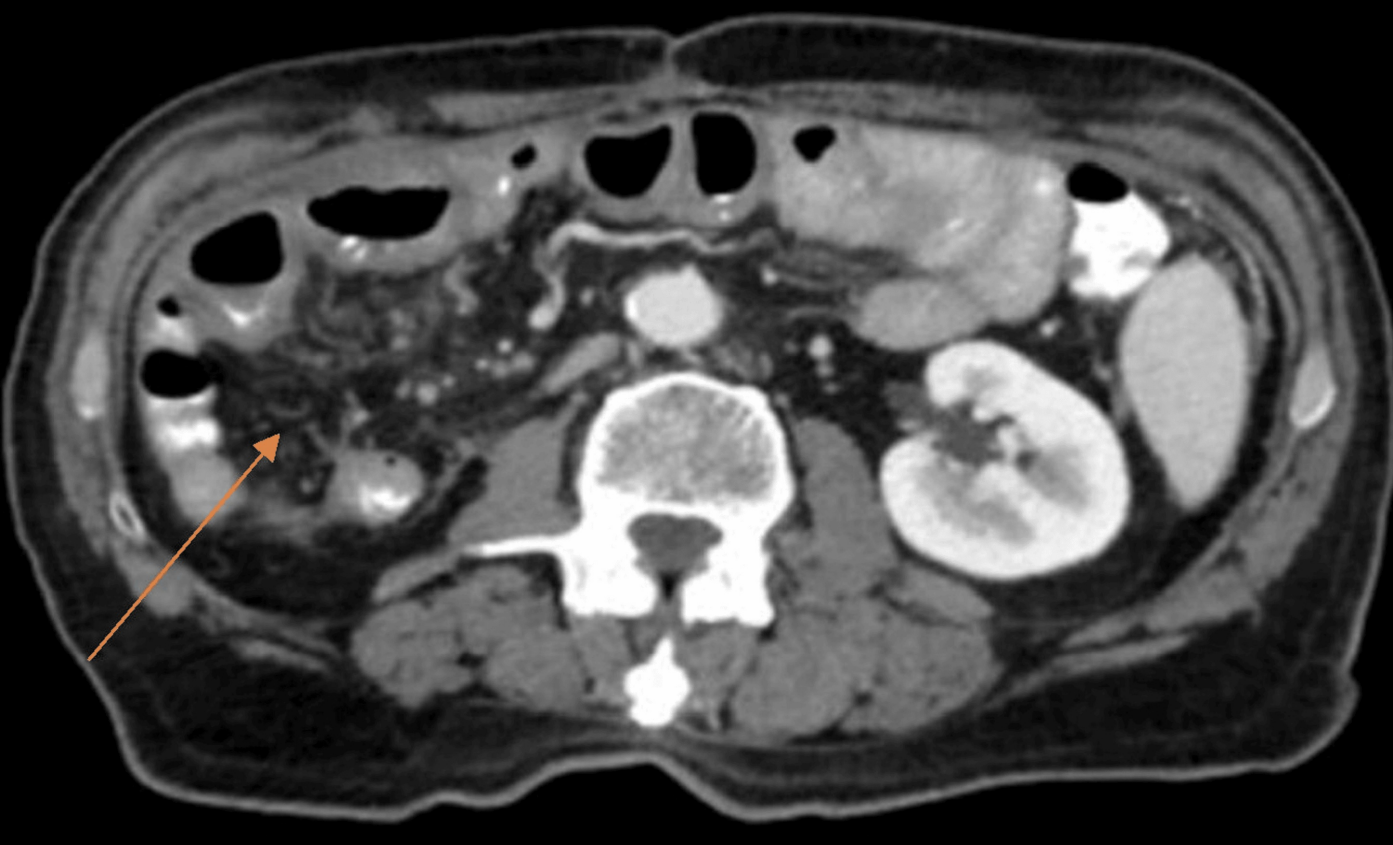
Axial computed tomography scan showing the absence of a solid tumor lesion, previously described (arrow)

In subsequent check-ups, a new abdominal and pelvic CT scan with oral and IV contrast was performed, showing adequate postoperative evolution, with no evidence of the previously described tumor, ascites, or adenomegaly in the retroperitoneum. No air-fluid levels were observed in the small intestine (Figure 4).

To date, the patient continues to undergo periodic check-ups by the Digestive Surgery and Oncology departments. He received adjuvant treatment with FOLFOX 6/6, with adequate clinical progress, no progression of the cancer, no palpable mass on physical examination, and is eating and performing activities without difficulty.

## Discussion

MCC is a rare neoplasm that differs from conventional adenocarcinoma in clinical, histological, and molecular aspects. Although classically described more frequently in elderly women and in the right colon [1,2], the patient in the case report was male with involvement of the ascending colon, which represents a less common location and highlights the possible anatomical heterogeneity in this entity. Histologically, MCC is characterized by solid sheets of large, poorly differentiated tumor cells with abundant eosinophilic cytoplasm, prominent nucleoli, and lymphocytic inflammatory infiltrate in the stroma [3,4]. The World Health Organization further notes that cells in MCC are arranged in sheets and lack the glandular structure characteristic of colon adenocarcinoma [4].

In this case, the initial diagnosis by endoscopic biopsy was poorly differentiated adenocarcinoma, requiring immunohistochemical study for definitive confirmation. This is consistent with reports in the literature, where the differential diagnosis can be complex and requires marker panels including CK20, CDX2, and MSI studies [5]. Typical immunohistochemical markers include positivity for CK20 and CDX2 in a subgroup of cases, as well as frequent negativity for CK7; however, the expression profile may vary and should be interpreted in conjunction with morphology [6,8]. Most MCCs have a strong association with MSI in up to 60% of cases, presenting as MSI-H and dMMR, and are associated with a better prognosis, although not all cases fit this profile. They generally exhibit positive staining for mucin 1 (MUC-1), mucin 2 (MUC-2), and trefoil factor 3 (TFF-3), indicating intestinal differentiation. They also characteristically show positive staining for calretinin and CDX2, and absence of MLH-1 [6]. Most cases reported in the literature show loss of both MLH1 and postmeiotic segregation increased 2 (PMS2) [7]. Our case presented with an isolated loss of MLH1 expression; PMS2 testing was not performed. The clinical presentation of MCC is nonspecific and overlaps with that of other colorectal cancers: abdominal pain, changes in bowel habits, anemia, or gastrointestinal bleeding [7]. The vegetative and friable lesion observed on colonoscopy was indicative of advanced neoplasia, but the location in the ascending colon and lymph node involvement (2/40) suggest a more reserved prognosis than in classic series, in which most tumors are right-sided and at an earlier stage [8].

The treatment of choice for MCC is oncological resection with adequate margins and regional lymphadenectomy, following the same principles applied to other colorectal adenocarcinomas [9]. In this context, right hemicolectomy was performed by exploratory laparotomy, with clear margins and adequate lymph node harvesting (40 nodes), which meets the oncological criteria recommended by the National Comprehensive Cancer Network (NCCN) and European Society for Medical Oncology (ESMO) guidelines [9,10]. Positive lymph node involvement justified the administration of adjuvant chemotherapy with the FOLFOX regimen, a strategy supported by studies suggesting benefit in patients with stage III disease or high-risk factors [11]. The evidence is not uniform, and there are reports of aggressive behavior, especially in cases with lymph node involvement or tumor deposits, such as the case referred to [12]. Therefore, it is important to provide close clinical and imaging follow-up to detect early recurrence in the short, medium, or long term.

## Conclusions

MCC is a rare disease with nonspecific symptoms that can be confused with other types of colorectal cancers. It therefore requires timely diagnosis, management, and treatment to reduce the recurrence rate. In this particular case, although there has been a favorable clinical evolution after specific surgical and oncological treatment, close clinical and imaging follow-up is required to detect early recurrences in the short, medium, or long term. Multicenter studies and national registries of this type of pathology could improve knowledge about its biological behavior and response to specific systemic treatments.
